# Dimensions of nursing-midwifery care during the COVID-19 pandemic in light of Jean Watson*

**DOI:** 10.1590/1980-220X-REEUSP-2024-0169en

**Published:** 2024-12-06

**Authors:** Juliana Amaral Prata, Jane Márcia Progianti, Camilla Ribeiro Freitas da Silva, Aline Caramez Costa, Luciane Marques de Araujo, Adriana Lenho de Figueiredo Pereira

**Affiliations:** 1 Universidade do Estado do Rio de Janeiro, Faculdade de Enfermagem, Departamento de Enfermagem Materno-Infantil, Rio de Janeiro, RJ, Brazil.; 2 Universidade do Estado do Rio de Janeiro, Programa de Pós-Graduação em Enfermagem, Rio de Janeiro, RJ, Brazil.

**Keywords:** Obstetric Nursing, Nursing Theory, Nursing Care, Childbirth, COVID-19, Enfermería Obstétrica, Teoría de Enfermería, Enfermería Cardiovascular, Parto, COVID-19

## Abstract

**Objective:**

To understand the theoretical dimensions of nursing-midwifery care for women in labor during the COVID-19 pandemic.

**Method:**

A qualitative study with 40 nurse-midwives. Data were collected from May to July 2021, through interviews, subjected to thematic content analysis and discussed in light of Jean Watson’s Theory of Transpersonal Human Caring.

**Results:**

The creative dimension encompassed the sixth, eighth and ninth elements of the process, manifesting itself in adaptations implemented in care. The humanistic and cultural dimensions were formed from the first, second, fourth and fifth elements, expressed in the way nurse-midwives care for, which subsidized the educational dimension, which encompassed the tenth element, as well as the spiritual dimension, elucidating the third element.

**Conclusion:**

The care provided by nurse-midwives to women in labor presented dimensions that permeated the ten elements of the Clinical Caritas process, enabling transpersonality in care in the face of COVID-19 control measures that, almost always, led to procedural, prescriptive and impersonal care.

## RESUMEN

**Objetivo:**

Comprender las dimensiones teóricas de la atención brindada por enfermeras obstétricas a las parturientas durante la pandemia de COVID-19.

**Método:**

Estudio cualitativo con 40 enfermeras obstétricas. Los datos se recopilaron de mayo a julio de 2021, a través de entrevistas, sometidas a análisis de contenido temático y discutidas a la luz de la Teoría del Cuidado Humano Transpersonal de Jean Watson.

**Resultados:**

La dimensión creativa abarcó los elementos sexto, octavo y noveno del proceso, manifestándose en las adaptaciones implementadas en el cuidado. Las dimensiones humanística y cultural se formaron a partir del primer, segundo, cuarto y quinto elementos, expresados en la forma de cuidar a las enfermeras obstétricas, que subvencionó la dimensión educativa, que abarcó el décimo elemento, así como la dimensión espiritual, dilucidando el tercer elemento.

**Conclusión:**

La atención brindada por enfermeras obstétricas a las parturientas presentó dimensiones que permearon los diez elementos del proceso de Cáritas Clínica, posibilitando la transpersonalidad en la atención frente a las medidas de control de la COVID-19 que, casi siempre, derivaron en asistencia procesal, prescriptiva e impersonal.

## INTRODUCTION

During the COVID-19 pandemic, obstetric services implemented strict disease control measures, including: prenatal consultations interspersed with teleconsultations; online educational groups; suspension of visits to the referral maternity ward; changes in the use of physical spaces and routines; use of PPE; reduction in circulation of people; physical distancing in care; definition of teams to care for positive cases; and restriction of companions and visitors during hospitalization^(1, 2, 3)^.

In this context, inadequacies in infrastructure associated with high demand for care, shortage of supplies and work overload created a challenging scenario for the preservation of good practices in childbirth care^(4,5)^. Thus, care for women during pregnancy and childbirth required resilience and creativity from professionals so that nurses adapted their practice to mitigate the risk of contamination, accommodate the demands of women in labor and ensure safe care, aligning it with updated scientific evidence^(6, 7, 8, 9)^.

From this perspective, it is worth highlighting that Jean Watson’s Theory of Transpersonal Human Caring offers an appropriate framework for understanding how these specialists acted to enrich their relationships with the people they assisted, develop awareness of caring for themselves and others, as well as create an environment of healing and care, restoring the well-being and meaning of life that is often lost^(10,11)^, especially in the face of suffering and deaths resulting from COVID-19.

This theory promotes comprehensiveness, enhances measures of comfort, well-being and pain control as well as the spiritual transcendence of suffering, as it understands that transpersonal nurses have the ability to center consciousness and intentionality on care, healing and wholeness, rather than pathology. To this end, Watson developed the Clinical Caritas process, composed of ten elements, envisioning it as a theoretical counterpoint to the term “curative”, which is dominant in medical science, whereas the term “caritas” means to care for, appreciate and give special attention^(10,11)^.

The first element refers to sustaining humanistic-altruistic values by practice of loving-kindness, compassion and equanimity with self/others. The second deals with being authentically present, enabling faith/hope/belief system, honoring subjective inner, life-world of self/others. The third concerns opening to spiritual, mystery, unknowns — allowing for miracles. The fourth highlights the commitment to developing and sustaining loving, trusting-caring relationships. The fifth concerns allowing for expression of positive and negative feelings — authentically listening to another person’s story. The sixth addresses creatively problem-solving-’solution-seeking’ through caring process; full use of self and artistry of caring-healing practices via use of all ways of knowing/being/doing/becoming. The seventh concerns engaging in transpersonal teaching and learning within the context of caring relationship; staying within other’s frame of reference; shift toward coaching model for expanded health/wellness. The eighth signals creating a healing environment at all levels, subtle environment for energetic authentic caring presence. The ninth discusses reverentially assisting with basic needs as sacred acts, touching mindbodyspirit of spirit of other; sustaining human dignity. Finally, the tenth element signals being sensitive to self and others by cultivating own spiritual practices; beyond ego-self to transpersonal presence^(10,12, 13, 14)^.

Given these theoretical notes and the adversities of the COVID-19 pandemic context, it is noted that nurse-midwives protected public policies aimed at women’s health, as they developed strategies to offer safe and humanized care to women in labor^(15,16)^. Thus, the present study aimed to understand the theoretical dimensions of nursing-midwifery care for women in labor during the COVID-19 pandemic.

## METHOD

### Study Design

This is descriptive and qualitative research, as it allows us to understand and describe the perceptions and opinions of a group about the phenomenon studied in a given context. The COnsolidated criteria for REporting Qualitative research (COREQ) guide recommendations, translated and validated for the Portuguese language, were followed.

### Location, Population and Selection Criteria

Nurses from different cities in the state of Rio de Janeiro who were specialists in nursing-midwifery and had been working in the care of women in labor for at least one year during the COVID-19 pandemic were included. Nurses who worked in the private sector were excluded, since the public sector is the main employer of nursing in Brazil, especially in the Southeast region, and in home birth services, since the publication of the Federal Nursing Council (In Portuguese, COFEN – *Conselho Federal de Enfermagem*) regulations for professional activity in this area was after the data collection period for this research.

### Data Collection

Data collection took place from May to July 2021, starting with the intentional recruitment of participants using snowball sampling, in which the first interviewee is called the seed, who nominates other people with the research profile, who nominates new participants until the sample reached saturation, when new nominations have already been interviewed or they do not provide new information^(17)^, which happened in the thirty-eighth interview, confirmed by carrying out two more.

The study included three seed participants (N1, N2 and N3), selected from the researchers’ contact with nursing-midwifery residency preceptors, due to their proximity to professionals who met the inclusion criteria. Three referral chains were established, in which there were 30 refusals, justified by work overload during the COVID-19 pandemic, and no withdrawals during data collection.

Potential participants were contacted via a messaging app to provide clarifications about the research, followed by an invitation to participate. Upon acceptance, the link to the Informed Consent Form (ICF) was shared in electronic form format. For health safety reasons, individual interviews took place via videoconference, using Google Meet^®^, on a date and time of professionals’ preference, and were mediated by two authors, residents in nursing-midwifery, who worked in childbirth care during data collection. They were previously trained and took turns conducting data collection.

To this end, a semi-structured script was prepared and divided into two parts: the first with closed-ended questions, for a brief intervieew characterization; and the second composed of two open-ended questions: how did you care for women in labor during the COVID-19 pandemic? What care did you provide? It is worth noting that three pilot tests were carried out, which comprised the analytical *corpus* of the study, as they confirmed the data collection instrument adequacy.

With authorization, the interviews took place in the presence of one of the authors and the professional, and were recorded with the support of an audio recorder application, lasting an average of 40 minutes. At the end, the material was transcribed using Microsoft Word^®^, totaling 238 pages. The transcribed content was sent for validation by participants, who did not respond with comments.

### Data Analysis and Processing

The data were subjected to thematic content analysis, involving three stages: pre-analysis; exploration and categorization; and data processing and interpretation^(18)^. In this process, skimming facilitated the identification of registration units (RUs) and context, followed by the selection of textual segments of interest, which were grouped according to semantic equivalence. This process resulted in the definition of four theoretical categories anchored in the elements of Jean Watson’s Clinical Caritas process, which were discussed in dialogue with the Theory of Transpersonal Human Caring and scientific productions on the subject.

These stages were developed by the two authors responsible for conducting the interviews and transcribing the material, and were validated by two other authors, professors with PhD and experience in qualitative research, through debriefing sessions to assess and interpret the findings.

### Ethical Aspects

The research complied with the ethical precepts of Resolution 466/2012 and Circular Letter 2/2021 of the Brazilian National Research Ethics Commission. It was approved by the *Universidade do Estado do Rio de Janeiro* Research Ethics Committee, under Opinion 4,518,637/2021 (02/01/2021). In compliance with standards for research involving human beings, participants were informed about the study objectives. They signed the ICF, and anonymity was ensured by using the letter “N”, referring to “nurse”, followed by a number referring to the order in which the interview was conducted.

## RESULTS

The study included 40 nurse-midwives, all female, the majority of whom were between 30 and 35 years old, obtained a specialist title through training in the form of residency and have an employment relationship under the CLT regimen. Regarding the length of experience in the specialty, 20 participants had between six and ten years, twelve had up to five years, and eight had more than ten years.

The thematic analysis of interviews culminated in four theoretical categories as follows: the first included six RUs and 40 text fragments, corresponding to the creative dimension of nursing-midwifery care; the second included three RUs and 34 text fragments, consistent with the humanistic and cultural dimensions of care; the third included two RUs and 19 text fragments, referring to the educational dimension of care; and the fourth included one RU and two text fragments, relating to the spiritual dimension of care.

### Creative Dimension of Care

This dimension was highlighted when nurse-midwives implemented strategies to respect health measures to control the disease, such as: replacing the shawl, which is a personal instrument of nurses, with the institution’s sheet to perform the rebozo; using gloves filled with heated water instead of the unit’s thermal bags; and sharing care with companions.

*When we need to do a rebozo, we use the unit’s sheet. After using it with the patient, we discard it in the hamper.* (N31)

*We have the thermal bags, but at the moment, I take the gloves, put hot water in them and use them as a compress because it’s easier to dispose of.* (N21)

*Some technologies, such as massage, we started to encourage companions to do more, because, before, we took turns to provide this care.* […] *now, we encourage those who are with her more to use this technology, to avoid proximity to her and contagion!* (N24)

Furthermore, they prioritized the provision of care instruments, such as the Swiss ball and two types of birthing stool, to women in labor who had a clear indication for their use, especially given the scarcity of this resource and the high demand for care. They also reduced the number of these instruments available in collective spaces and encouraged their use in individual spaces.

*In the space where the* [Swiss] *ball is, we reduced the number so that there are not too many women together.* […] *not to let them be crowded, but they continue to have access to non-invasive care technologies.* (N5)

*We try to offer her* [instruments] *inside the box, but with precautions due to the pandemic, to avoid circulation.* […] *a birthing stool, a ball, individually. We offer music, we try to work with her psychologically because that is her moment, regardless of this pandemic!* (N19)

*We assess who needs* [the instruments] *the most.* […] *we try to get a little closer!* (N26)

*We only have a birthing stool, two stools and a ball.* […] *we end up taking them from one, cleaning them and taking them to another.* (N36)

Nurses were also concerned with the care environment, in order to meet the needs of women in labor and respect their right to the presence of a companion, freedom of movement, privacy and autonomy, with attention to the temperature, lighting and sounds of the physical space in which they were located.

*Nursing-midwifery has this role amidst the pandemic… to provide the woman in labor with a respectful environment, where she feels good, is in control of her birth, with the right to a companion… even in the case of a COVID-positive patient, giving the right to have a companion and providing this freedom, adopting protective measures.* (N23)

*We use aromatherapy, chromotherapy… being attentive to listening, being alone when needed and the environment, which we try to keep silent, as welcoming as possible, at the temperature that she feels comfortable at and with the lighting she chooses, whether bright or dim.* (N25)

### Humanistic and Cultural Dimensions of Care

This dimension was highlighted when, even in the face of the adversities of the pandemic context, nurse-midwives protected their way of acting in caring for women in labor, as they understood the importance of mitigating fear, respecting cultural values, identifying needs and providing a relaxing environment, through welcoming, building bonds, active listening, demonstrations of affection and respect, encouraging women’s confidence and providing guidance.

*We need to work on women’s confidence during pregnancy and labor,* […] *to be able to provide a little more security, because they are already afraid of the pain of childbirth and, now, the fear of a pandemic!* (N8)

*They arrive at the maternity ward to give birth, afraid, not knowing what will happen* […] *they have this fear of the unknown, that cultural thing that childbirth is suffering* […]. *We will all feel pain when giving birth, but we are the ones who bring the suffering to the birth. I continue to work with women on this, regardless of whether it is a pandemic or not.* (N5)

*First, it is about listening to the woman, knowing her needs. There is no point in us talking if she doesn’t understand. It is about knowing her needs and listening, with respect and affection. Then, guide her according to her needs.* (N30)

*Of all the technologies we have, one of the most important is active listening.* […] *when you offer her a listening ear, you guide her through her labor very well and she creates a bond with you, she will be able to relax much more!* (N6)

Furthermore, they were sensitive to promoting comfort during labor, respecting the desire of women in labor to remove their masks, as well as encouraging freedom of movement, observing the signs shown by the woman’s body to propose care.

*During the expulsion, they take off their masks. Some professionals ask them to put the mask on right away. I can’t have that approach during expulsion!* (N22)

*During labor, in the active phase, I let them take off the mask to be more comfortable because we know it is difficult for them.* (N27)

*We notice, by the movement of the woman’s body… if she is making a lot of pelvic movement and we ask her if she doesn’t want to try using the ball, if it makes her more comfortable.* […] *we take advantage of the moment of the woman’s response, of her body, of the spontaneous movement…* (N12)

### Educational Dimension of Care

This dimension of care was highlighted when nurse-midwives provided teaching and learning spaces based on dialogue, sharing of knowledge and experiences, providing information about the birthing process and providing guidance on care possibilities, with respect for the autonomy of women in labor.

*Explaining the physical space… this type of conversation helps, because visits to the maternity ward are blocked due to the pandemic.* […] *I always encourage people to talk about my own experience of childbirth.* […] *the information that she can walk, the positions that exist, that changing positions throughout labor helps.* (N3)

*Knowing what information she has to try to, at least, make her feel safer, having the information. In my opinion, if I can reach her, I think it will be a great gain for her and for me, because I will be happy to see that she is able to understand everything that is happening during her hospitalization.* (N2)

*I don’t impose on women what they have to do. I like to ask them if they want to try and experiment, because we have to let them be free. For some women, I’ll need to suggest something.* […] *I try to talk and give them guidance.* […] *we always have to guide them so that they act in the way that makes them feel best and give them options.* (N15)

### Spiritual Dimension of Care

This theoretical category was revealed when nurse-midwives understood the influence of religiosity on the childbirth experience, respected the manifestation of beliefs of women in labor and took a stance in defense of judgment-free care.

*Everyone has their own religion and sometimes it ends up overflowing* […]. *I always try to respect women’s space, not exposing myself as a religion because the State is secular and we have to respect the differences of others, the culture of others… I’ve seen patients with ribbons on their belly.* […] *we respect them!* […] *each professional needs to know how to deal with this situation, with the differences, with each person’s religion and culture.* (N5)

*We have religious issues present at the time of childbirth. It is extremely important for some women!* […] *we have to respect any manifestation on their part!* […] *I notice some judgments that I do not like and I point out that assistance needs to be free of judgments. It needs to be the same for everyone!* (N32)

## DISCUSSION

The adaptations implemented by nurse-midwives in caring for women in labor, in light of COVID-19 control measures, reveal the creative dimension of care, which encompassed the following elements of the Clinical Caritas process: creatively problem-solving-’solution-seeking’ through caring process; full use of self and artistry of caring-healing practices via use of all ways of knowing/being/doing/becoming (sixth element); creating a healing environment at all levels, subtle environment for energetic authentic caring presence (eighth element); and reverentially assisting with basic needs as sacred acts, touching mindbodyspirit of spirit of other; sustaining human dignity (ninth element).

Creativity, understood as the ability to think differently, be innovative and create new things, associated with proactivity, which refers to the initiative to act beyond prescribed activities, configured fundamental skills for health work during the pandemic, which favored the identification of opportunities, facing the unknown and adjustments to the accelerated pace of change^(19)^.

Thus, the health crisis awakened nursing’s ability to reinvent itself, as evidenced among the nurse-midwives in this study, who developed care actions in line with the protocols for reorganizing health services during the pandemic, which signaled the need to avoid sharing objects, such as those adopted for pain relief, reduce circulation of people in health services and maintain physical distancing in care^(2, 3, 4, 5)^.

As identified in other research, nurse-midwives mobilized to maintain an adequate environment, promote comfort and well-being, encourage the physiology of parturition and ensure women’s rights during labor and birth, without losing sight of the safety aspects of the care process given the limitations imposed by inadequacies in the physical structure of services and the shortage of human and material resources^(1,6,9,16,20)^.

On the other hand, these adaptations are also aligned with the sixth element of Jean Watson’s Clinical Caritas process, as nurses applied all available knowledge and used creative imagination associated with scientific logic to formulate proposals for solving problems and, thus, make adaptations in the care process, resorting to different ways of knowing/being/doing/becoming and considering the specificities of a person in their entirety^(10,12, 13, 14)^.

The eighth element was manifested in nurses’ intentional actions to provide a care environment that was conducive to comfort, relaxation, privacy and safety while respecting the preferences of women in labor regarding temperature, lighting and sound. This finding reveals participants’ concern with the harmony of a person’s mind, body and soul with the external environment, understanding that this alignment positively affects physical and emotional health, facilitating interpersonal relationships and enabling a feeling of satisfaction with life^(10,21)^.

Furthermore, in meeting the basic needs presented by women during the birthing process, nurses demonstrated an intentionally careful awareness to understand the dynamics of subjectivities in the pandemic context and protect human dignity^(12,22)^, thus revealing the ninth element of the Clinical Caritas process.

In addition to these aspects, welcoming, establishing a bond and building a relationship of respect, affection and trust were valued by participants in the process of caring for women in labor, revealing the humanistic and cultural dimensions of care by nurse-midwives, which were anchored in the following elements of the Clinical Caritas process: manifesting humanistic, altruistic and ethical values about oneself and others (first element); having an authentic presence that respects faith, hope, belief and subjectivities (second element); committing to building relationships of care based on love and trust (fourth element); and allowing the expression of feelings through authentic listening to the other (fifth element).

Welcoming is a process that involves actions to favor the establishment of a bond, trust and commitment between health professionals and users, enabling the identification of their needs and the offering of humanized and effective care^(12,21,22)^. However, the COVID-19 pandemic caused significant changes in physical facilities and institutional routines, which directly affected welcoming and humanization protocols in favor of collective safety^(23,24)^.

In this context, nurse-midwives understood that the social and emotional issues inherent to pregnancy during the pandemic, associated with hospitalization for childbirth, are factors that generate anguish, fear, and discomfort that can significantly influence the experience of childbirth. Therefore, they showed empathy and developed sensitive and respectful care to ensure bond, demonstrate support, transmit security, promote well-being, and create the necessary conditions for the exercise of autonomy, even in the face of adversities faced in obstetric services^(16,24)^.

Based on these concepts, nurse-midwives loosen some measures established in COVID-19 control protocols in an attempt to align institutional routines with the needs and subjectivities of women in labor, as identified in other studies^(5,7)^. In this regard, through observation and sensitivity, they offered care to promote comfort and physiological evolution of parturition, such as allowing women to remove their masks during the expulsive period of labor and providing free movement during labor.

Encouraging deep, conscious breathing during labor reduces circulation of stress-related hormones and increases the release of endorphins and oxygen levels, reducing blood pressure and making the sensations of labor more pleasant. Furthermore, freedom of movement during labor improves uterine dynamics, increases pain tolerance, optimizes cervical dilation and fetal presentation, favors sacral mobility, increases the anteroposterior and transverse diameters of the pelvis, and contributes to maternal pushing, fetal oxygenation, and perineal integrity^(25)^.

It is worth noting that, during the pandemic, both practices were hampered by recommendations regarding the use of masks by women in care settings, since deep breathing and expulsive efforts increased exposure to respiratory secretions and given the need to avoid circulation of people and maintain physical distancing to mitigate COVID-19 transmission. However, it is considered that the use of a protective mask can interfere with the gas and metabolic exchanges inherent to the physiology of labor, exacerbate respiratory conditions, compromise communication and cause a sensation of discomfort and overheating, whereas the prohibition of free movement may constitute a violation of women’s rights during childbirth^(9,16)^.

Therefore, the characteristics of the process of caring for nurse-midwives with women in labor during the pandemic reveal convergence with the first and fourth elements of the Clinical Caritas process, since nurses’ dialogue, listening and demonstrations of empathy, respect, availability, proximity, sensitivity and involvement provide opportunities for sharing life stories, desires and anxieties, contributing to relationships of kindness, help and trust and shaping transpersonal care aimed at promoting the comfort, well-being and recovery of the person being cared for. From this perspective, nurses enter into the experience of care and vice versa, connecting and becoming aware of the problems of others in a way that transcends the physical aspect^(10,12, 13, 14,26)^.

From this perspective, the second and third elements of the process were highlighted, since participants in this study: demonstrated intentional awareness and authentic presence; remained attentive to subjectivities; recognized women in their entirety; perceived their movements and meanings; enabled the free expression of thoughts and feelings; were respectful and understanding; and encouraged trust and hope in a scenario of widespread fear and uncertainty^(10,26,27)^.

Permeating the care of women in labor during the pandemic, the educational dimension of care provided by participants stands out, as they showed themselves to be genuinely engaged in teaching and learning actions. Thus, the care added transpersonal characteristics, since, from the encounter, nurses establish an intersubjective relationship that requires authenticity, being reflexively present for oneself and for the other and acting with an intentional awareness to promote exchange of knowledge, experiences and meanings, in addition to capturing a person’s reality, perceiving how they understand and respond to the circumstances of a given moment^(10,12,13,14)^.

In this process concerning the tenth element of the Clinical Caritas process, nurses establish an empathetic relationship and recognizes a person’s right to choose; therefore, they consider their knowledge, experiences and needs, provide appropriate information, clarify doubts and encourage their participation in decision-making processes, demonstrating respect for their references of beliefs and values^(11,14)^. From this perspective, nurse-midwives offer guidance and care options, as they understand that dialogue provides opportunities for adapting the care process to the cultural reality of women in labor, providing respectful care that generates trust and favors adherence to the proposed care^(25)^, as identified among participants in this research.

It is known that, during the COVID-19 pandemic, the dialogic proposal of health education activities developed by nurses was fundamental to disseminate science-based information, encourage safe behaviors, promote self-care and minimize suffering related to the high numbers of contamination and death from the disease^(28)^. In this context, religiosity and spirituality were also coping resources used by many people, since they encourage care, generosity, empathy, solidarity and resilience as expressions of life based on humanistic principles^(29)^.

It is important to clarify that religiosity deals with rituals and ceremonies related to the transcendent, practiced in a private or public environment, contemplating specific beliefs about life, death, roles and conduct in the context of certain social groups. On the other hand, spirituality refers to the personal search for the meaning of life and its relationship with the sacred and the transcendent, which enables transforming a person’s reality, regardless of association with a religion or community^(30)^. Hence, both are related to increased quality of life, improved immune function and vigor in people with chronic diseases, reduced stress related to degenerative and neurological pathologies as well as reduced hospital stay^(29,30)^.

From this recognition, it is noted that the process of caring for nurse-midwives contemplated a spiritual dimension, which helps in self-discovery, in maintaining health and in facing situations considered difficult^(10,14)^. From this perspective, the third element of the Clinical Caritas process is identified, which translates into nurses’ sensitivity and commitment to a person’s faith-hope system and care provision aimed at reestablishing vital balance, rescuing the meanings of life and self-realization. At the same time, the tenth element was presented, relating to the expansion of nurses’ vision of spiritual and unknown phenomena, enabling the preservation of meanings, beliefs, myths, metaphors and the world of inner and subjective life, through care that encompasses the totality of the being and transcends the linearity of doing^(10,12,13,14,27)^.

Considering the above, it is worth highlighting that the abstract aspects that make up Jean Watson’s proposal for transpersonal care and the fact that it is a far-reaching theory may make it difficult to apply in nursing practice^(14,21)^. However, even though the nurse-midwives in this study did not use the aforementioned theory to systematize their care amid the adversities of the COVID-19 pandemic, it was possible to perceive that the dimensions of care provided by these specialists present aspects that refer to the ten elements of the Clinical Caritas process.

As limitations of this study, we pointed out the technique adopted to recruit nurse-midwives, which may have resulted in a certain homogeneity of participants, who worked in public childbirth care services in Rio de Janeiro. Therefore, the findings of this research are not representative of the reality experienced by nurses in other Brazilian locations during the pandemic context.

## CONCLUSION

It is evident that, during the COVID-19 pandemic, the care provided by nurse-midwives to women in labor presented dimensions that crossed the ten elements of Jean Watson’s Clinical Caritas process. Therefore, they were able to access the subjective aspects of women in labor to implement transpersonality in care, helping them achieve a higher degree of harmony, in an environment where the COVID-19 control measures implemented in health services led to procedural, prescriptive, curative and impersonal care. These findings point to the relevance of the work of these specialists in promoting humanization and good practices even in scenarios of health crisis, and studies are needed that explore women’s perspectives on labor and birth care in this context.
