# Supplementary material for: Dimensions of nursing-midwifery care during the COVID-19 pandemic in light of Jean Watson
Source: Rev Esc Enferm USP. 2024 Dec 6;58:e20240169. doi: 10.1590/1980-220X-REEUSP-2024-0169en (PMC11627506; doi:10.1590/1980-220X-REEUSP-2024-0169en)
Supplement: Translation

# Translation: Dimensões do cuidado das enfermeiras obstétricas na pandemia de COVID-19 à luz de Jean Watson*

**DOI:** 10.1590/1980-220X-REEUSP-2024-0169pt

**Authors:** Juliana Amaral Prata, Jane Márcia Progianti, Camilla Ribeiro Freitas da Silva, Aline Caramez Costa, Luciane Marques de Araujo, Adriana Lenho de Figueiredo Pereira

**Affiliations:** 1 Universidade do Estado do Rio de Janeiro, Faculdade de Enfermagem, Departamento de Enfermagem Materno-Infantil, Rio de Janeiro, RJ, Brasil.; 2 Universidade do Estado do Rio de Janeiro, Programa de Pós-Graduação em Enfermagem, Rio de Janeiro, RJ, Brasil.

**Keywords:** Enfermagem Obstétrica, Teoria de Enfermagem, Enfermagem Cardiovascular, Parto, COVID-19

## Abstract

**Objetivo:**

Compreender as dimensões teóricas do cuidado das enfermeiras obstétricas às parturientes durante a pandemia de COVID-19.

**Método:**

Estudo qualitativo com 40 enfermeiras obstétricas. Os dados foram coletados de maio a julho de 2021, através de entrevistas, submetidos à análise de conteúdo temática e discutidos à luz da Teoria do Cuidado Humano Transpessoal de Jean Watson.

**Resultados:**

A dimensão criativa abarcou o sexto, oitavo e nono elementos do processo, manifestando-se nas adaptações implementadas no cuidar. As dimensões humanística e cultural se conformaram a partir do primeiro, segundo, quarto e quinto elementos, expressas no modo de cuidar das enfermeiras obstétricas, que subsidiou a dimensão educativa, que abrangeu o décimo elemento, bem como a dimensão espiritual, elucidando o terceiro elemento.

**Conclusão:**

O cuidado das enfermeiras obstétricas às parturientes apresentou dimensões que perpassaram pelos dez elementos do processo *Clinical Caritas*, possibilitando a transpessoalidade no cuidar diante das medidas de controle da COVID-19 que, quase sempre, conduziam a uma assistência procedimental, prescritiva e impessoal.

## INTRODUÇÃO

Durante a pandemia de COVID-19, os serviços obstétricos implementaram medidas rigorosas de controle da doença, incluindo: consulta pré-natal intercalada com teleconsulta; grupos educativos *online*; suspensão das visitas à maternidade de referência; mudanças no uso dos espaços físicos e nas rotinas; utilização de EPI; redução da circulação de pessoas; distanciamento físico nos cuidados; definição de equipes para o atendimento dos casos positivos; e restrição de acompanhantes e visitas durante a internação hospitalar^(1,2,3)^.

Nesse panorama, as inadequações na infraestrutura associadas à alta demanda assistencial, à escassez de insumos e à sobrecarga laboral conformaram um cenário desafiador para a preservação das boas práticas na atenção ao parto e nascimento^(4,5)^. Assim, a assistência às mulheres no ciclo gravídico-puerperal demandou resiliência e criatividade dos profissionais, de modo que as enfermeiras adaptaram sua prática para mitigar o risco de contaminação, acolher as demandas das parturientes e assegurar cuidados seguros, alinhando-a com as evidências científicas atualizadas^(6,7,8,9)^.

Sob essa ótica, salienta-se que a Teoria do Cuidado Humano Transpessoal de Jean Watson oferece um arcabouço apropriado para compreender como essas especialistas agiram no sentido de enriquecer suas relações com as pessoas assistidas, desenvolver a consciência do cuidado de si e do outro, bem como criar um ambiente de cura e cuidados, restituindo o bem-estar e o sentido da vida que muitas vezes se perde^(10,11)^, especialmente diante dos sofrimentos e das mortes decorrentes da COVID-19.

Essa teoria impulsiona a integralidade, potencializa as medidas de conforto, bem-estar e controle da dor, bem como a transcendência espiritual do sofrimento, pois compreende que a enfermeira transpessoal tem a capacidade de centrar a consciência e a intencionalidade no cuidado, na cura e na totalidade, em detrimento da patologia. Para tanto, Watson elaborou o processo *Clinical Caritas*, composto por dez elementos, vislumbrando-o como um contraponto teórico ao termo “curativo”, que é dominante na ciência médica, ao passo que o termo “caritas” significa cuidar, apreciar e dar atenção especial^(10,11)^.

O primeiro elemento se refere aos valores humanístico, altruísta e ético manifestados pela enfermeira em relação a si mesma e aos outros. O segundo trata da presença autêntica que respeita a fé, a esperança, a crença e as subjetividades. O terceiro diz respeito ao ser sensível aos valores e práticas espirituais. O quarto destaca o empenho na construção de relações de cuidado baseadas na amorosidade e confiança. O quinto permite a expressão de sentimentos por meio da escuta autêntica da história do outro. O sexto aborda a resolução criativa dos problemas. O sétimo compreende a relação de cuidado como um processo dialógico de ensino e aprendizagem. O oitavo sinaliza a criação de um ambiente de cura que favoreça a presença energética, autêntica e carinhosa. O nono discorre sobre o atendimento das necessidades básicas, com respeito à dignidade humana. E, por fim, o décimo elemento sinaliza a importância da intuição^(10,12,13,14)^.

Diante desses apontamentos teóricos e das adversidades do contexto pandêmico da COVID-19, nota-se que as enfermeiras obstétricas resguardaram as políticas públicas voltadas para a saúde das mulheres, pois desenvolveram estratégias para oferecer cuidados seguros e humanizados às parturientes^(15,16)^. Assim, o presente estudo objetivou compreender as dimensões teóricas do cuidado das enfermeiras obstétricas às parturientes durante a pandemia de COVID-19.

## MÉTODO

### Tipo de Estudo

Trata-se de pesquisa descritiva e qualitativa, pois este tipo de estudo permite apreender e descrever as percepções e opiniões de um grupo acerca do fenômeno estudado em um determinado contexto. Foram seguidas as recomendações do guia *COnsolidated criteria for REporting Qualitative research* (COREQ), versão traduzida e validada para a língua portuguesa.

### Local, População e Critérios de Seleção

Incluíram-se no estudo enfermeiras de diferentes municípios do estado do Rio de Janeiro que eram especialistas em enfermagem obstétrica e atuavam na assistência às parturientes por, pelo menos, um ano durante o curso pandêmico da COVID-19. Excluíram-se aquelas que atuavam na rede privada, visto que o setor público é o principal empregador da enfermagem no Brasil, sobretudo na região Sudeste, e em serviços de parto domiciliar, pois a publicação das normativas do Conselho Federal de Enfermagem (COFEN) para a atuação profissional nesse âmbito foi posterior ao período de coleta de dados desta pesquisa.

### Coleta de Dados

A coleta de dados aconteceu de maio a julho de 2021, iniciando com a captação intencional dos participantes por meio da técnica de bola de neve, em que o primeiro entrevistado é denominado de semente, que indica outras pessoas com o perfil da pesquisa, que indicam novos participantes até o alcance da saturação da amostra, quando as novas indicações já foram entrevistadas ou as mesmas não fornecem informações inéditas^(17)^, o que aconteceu na trigésima oitava entrevista, confirmada com a realização de outras duas.

O estudo contou com três participantes sementes (E1, E2 e E3), selecionados a partir do contato das pesquisadoras com preceptores da residência em enfermagem obstétrica, pela proximidade com profissionais que atendiam aos critérios de inclusão. Constituíram-se três cadeias de indicação, nas quais tiveram 30 recusas, justificadas pela sobrecarga de trabalho durante a pandemia de COVID-19, e nenhuma desistência ao longo da coleta dos dados.

O contato com os participantes potenciais aconteceu por meio de um aplicativo de mensagem, para esclarecimentos sobre a pesquisa, seguido do convite para participação. Mediante o aceite, compartilhou-se o *link* do Termo de Consentimento Livre e Esclarecido (TCLE) no formato de formulário eletrônico. Por questões de segurança sanitária, as entrevistas individuais aconteceram por videoconferência, utilizando a plataforma *Google Meet* ^®^, em data e horário de preferência do profissional, e foram mediadas por duas autoras, residentes em enfermagem obstétrica, que atuavam na assistência ao parto durante a coleta de dados, as quais foram previamente treinadas e se revezaram na condução da coleta dos dados.

Para tanto, foi elaborado um roteiro semiestruturado, dividido em duas partes: a primeira com perguntas fechadas, para uma breve caracterização dos entrevistados; e a segunda composta por duas questões abertas: como você cuidou das parturientes durante a pandemia de COVID-19? Quais cuidados você desenvolveu nessa assistência? Destaca-se que foram realizados três testes piloto, os quais compuseram o *corpus* analítico do estudo, pois confirmaram a adequação do instrumento de coleta dos dados.

Mediante a autorização, as entrevistas transcorreram na presença de uma das autoras e do profissional, sendo gravadas com o apoio de um aplicativo de gravador de áudio, durando em média 40 minutos. Ao término, procedeu-se à transcrição do material que, utilizando o *software Microsoft Word* ^®^, totalizou 238 páginas. O conteúdo transcrito foi encaminhado para a validação dos participantes, que não retornaram com comentários.

### Análise e Tratamento dos Dados

Os dados foram submetidos à análise de conteúdo temática, envolvendo três etapas: pré-análise; exploração e categorização; e tratamento e interpretação dos dados^(18)^. Nesse percurso, a leitura flutuante favoreceu a identificação das unidades de registro (UR) e de contexto, seguida da seleção dos segmentos textuais de interesse, que foram agrupados de acordo com a equivalência semântica. Esse processo resultou na definição das quatro categorias teóricas ancoradas nos elementos do processo *Clinical Caritas* de Jean Watson, que foram discutidas em diálogo com a Teoria do Cuidado Humano Transpessoal e produções científicas sobre o tema.

Essas etapas foram desenvolvidas pelas duas autoras responsáveis pela realização das entrevistas e transcrição do material, sendo validadas por outras duas autoras, professoras com doutorado e experiência em pesquisas qualitativas, por meio de sessões de *debriefing* para avaliação e interpretação dos achados.

### Aspectos éticos

A pesquisa respeitou os preceitos éticos da Resolução nº 466/2012 e do Ofício Circular nº 2/2021 da Comissão Nacional de Ética em Pesquisa. Obteve a aprovação do Comitê de Ética em Pesquisa da Universidade do Estado do Rio de Janeiro, sob Parecer n° 4.518.637/2021 (01/02/2021). Em atendimento às normas para pesquisas envolvendo seres humanos, os participantes foram esclarecidos quanto aos objetivos do estudo. Eles assinaram o TCLE, e o anonimato foi assegurado por meio da utilização da letra “E”, concernente à “enfermeira”, acompanhada de um algarismo referente à ordem de realização da entrevista.

## RESULTADOS

Participaram do estudo 40 enfermeiras obstétricas, todas do sexo feminino, sendo que a maioria se encontra na faixa etária entre 30 e 35 anos, obteve o título de especialista por meio da formação na modalidade de residência e possui vínculo empregatício do tipo celetista. Em relação ao tempo de atuação na especialidade, 20 participantes têm entre seis e dez anos, doze, até cinco anos, e oito, mais de dez anos.

A análise temática das entrevistas culminou em quatro categorias teóricas, sendo que: a primeira abarcou seis UR e 40 fragmentos de texto, correspondentes à dimensão criativa do cuidado das enfermeiras obstétricas; a segunda agregou três UR e 34 fragmentos de texto, consoantes com as dimensões humanística e cultural do cuidado; a terceira incluiu duas UR e 19 fragmentos de texto, referentes à dimensão educativa do cuidado; e a quarta abrangeu uma UR e dois fragmentos de texto, relativos à dimensão espiritual do cuidado.

### Dimensão Criativa do Cuidado

Essa dimensão foi evidenciada quando as enfermeiras obstétricas implementaram estratégias para respeitar as medidas sanitárias de controle da doença, tais como: substituíram o xale, que é um instrumento pessoal da enfermeira, pelo lençol da instituição para a realização do rebozo; utilizaram a luva preenchida com água aquecida ao invés das bolsas térmicas da unidade; e compartilharam o cuidado com os acompanhantes.

*Quando a gente precisa fazer rebozo, usa o lençol da unidade. Depois de utilizar com a paciente, descarta no hamper.* (E31)

*Temos as bolsinhas térmicas, mas, no momento, eu pego as luvas, coloco água quente e uso como compressa para elas porque é mais fácil de descartar.* (E21)

*Algumas tecnologias, como a massagem, a gente passou a incentivar mais o acompanhante para fazer, porque, antes, a gente revezava muito esse cuidado.* […] *agora, a gente incentiva mais quem está com ela para aplicar essa tecnologia, para evitar a proximidade com ela e o contágio!* (E24)

Além disso, priorizaram a oferta de instrumentos do cuidar, como a bola suíça, o cavalinho e a banqueta de parto, às parturientes que apresentavam indicação clara para o uso, sobretudo diante da escassez desse recurso e da alta demanda assistencial. Ainda, reduziram o quantitativo desses instrumentos disponibilizados nos espaços coletivos e incentivaram a utilização em espaços individuais.

*No espaço onde tem a bola* [suíça], *reduzimos o número para que não fiquem muitas mulheres juntas.* […] *não deixar que elas fiquem aglomeradas, mas elas continuam tendo acesso às tecnologias não invasivas de cuidado.* (E5)

*A gente tenta oferecer* [instrumentos] *para ela dentro do box, mas com os cuidados devido à pandemia, para evitar a circulação.* […] *um cavalinho, uma bola, individualmente. Oferece música, tenta trabalhar com ela psicologicamente porque ali é o momento dela, independente dessa pandemia!* (E19)

*A gente avalia quem mais precisa* [dos instrumentos]. […] *procura ficar um pouco mais próximo!* (E26)

*Só temos um cavalinho, duas banquetas e uma bola*. […] *Acaba que tiramos de uma, limpamos e levamos para outra.* (E36)

As enfermeiras também se preocuparam com o ambiente do cuidado, no sentido de atender às necessidades das parturientes e respeitar o seu direito à presença do acompanhante, à liberdade de movimentos, à privacidade e à autonomia, com atenção à temperatura, iluminação e sons do espaço físico no qual ela se encontrava.

*A enfermagem obstétrica tem esse papel em meio a pandemia… de proporcionar para a parturiente um ambiente respeitoso, onde ela se sinta bem, seja dona do seu parto, com direito a um acompanhante… mesmo em caso de paciente com COVID positivo, dar o direito de ter um acompanhante e proporcionar essa liberdade, adotando as medidas de proteção.* (E23)

*A gente usa a aromaterapia, cromoterapia… estar atenta à escuta, ficar sozinha quando precisa e ao ambiente, que a gente tenta deixar silencioso, o mais acolhedor possível, na temperatura que ela sente conforto e com a iluminação que ela escolhe, seja claro ou na penumbra.* (E25)

### Dimensões Humanística e Cultural do Cuidado

Essa dimensão foi evidenciada quando, mesmo diante das adversidades do contexto pandêmico, as enfermeiras obstétricas resguardaram o modo de agir no cuidado às parturientes, visto que compreenderam a importância de mitigar o medo, respeitar valores culturais, identificar as necessidades e proporcionar um ambiente de relaxamento, por meio do acolhimento, da construção do vínculo, da escuta ativa, das demonstrações de afeto e respeito, do incentivo à confiança da mulher e orientações.

*A gente precisa trabalhar na mulher a confiança na gestação e durante o trabalho de parto,* […] *conseguir levar um pouco mais de segurança, porque ela já tem o medo da dor de parir e, agora, o medo de uma pandemia!* (E8)

*Elas chegam na maternidade para parir, com medo, sem saber o que vai acontecer* […] *elas têm esse medo do desconhecido, aquela coisa cultural de que parto é sofrimento* […]. *A dor, todas nós vamos sentir para parir, mas, o sofrimento, somos nós que trazemos para o parto. Isso eu continuo trabalhando com as mulheres, independente de estar na pandemia ou não.* (E5)

*Primeiro, é ouvir a mulher, saber das necessidades dela. Não adianta a gente falar e ela não assimilar. É saber a necessidade e escutar, com respeito e carinho. Depois, orienta de acordo com a necessidade dela.* (E30)

*De todas as tecnologias que a gente tem, uma das principais é a escuta ativa.* […] *quando você oferece uma escuta para ela, orienta muito bem o trabalho de parto e ela cria o vínculo com você, ela vai conseguir relaxar muito mais!* (E6)

Além disso, se mostraram sensíveis à promoção do conforto durante o trabalho de parto, respeitando o desejo das parturientes de retirar a máscara, bem como ao incentivo à liberdade de movimentos, observando os sinais manifestados pelo corpo da mulher para propor cuidados.

*No expulsivo, elas tiram a máscara. Tem profissional que pede para botar a máscara na hora. Eu não consigo ter essa abordagem na hora do expulsivo!* (E22)

*No momento de trabalho de parto, na fase ativa, eu deixo elas tirarem a máscara para ficarem mais confortáveis porque a gente sabe que é difícil para elas.* (E27)

*A gente percebe, pela movimentação do corpo da mulher… se ela está fazendo bastante movimento pélvico e perguntamos se ela não quer tentar utilizar a bola, se fica mais confortável.* […] a *gente aproveita o momento de resposta da mulher, do corpo dela, da movimentação espontânea…* (E12)

### Dimensão Educativa do Cuidado

Essa dimensão do cuidado foi evidenciada quando as enfermeiras obstétricas oportunizaram espaços de ensino e aprendizagem baseados no diálogo, no compartilhamento de saberes e experiências, no fornecimento de informações sobre o processo de parturição e nas orientações acerca das possibilidades de cuidado, com respeito à autonomia das parturientes.

*Explicar o espaço físico… esse tipo de conversa ajuda, porque a visita à maternidade está bloqueada por conta da pandemia.* […] *eu sempre estimulo em relação a minha experiência própria de parto.* […] *a informação que ela pode caminhar, as posições que existem, que mudar de posição ao longo do trabalho parto ajuda.* (E3)

*Saber o que ela tem de informação para tentar, pelo menos, fazer com que ela, tendo a informação, se sinta mais segura. Na minha fala, se eu conseguir alcançá-la, eu acho que já é um grande ganho para ela e para mim, porque eu vou ficar feliz de ver que ela está conseguindo entender tudo o que está acontecendo durante a internação.* (E2)

*Eu não imponho à mulher o que ela tem que fazer. Eu gosto de perguntar se ela quer tentar e experimentar, porque temos que deixá-las livres. Algumas mulheres, eu vou precisar sugerir alguma coisa.* […] *eu tento conversar e vou orientando.* […] *a gente tem sempre que orientar para que ela aja do modo que se sinta melhor e dar opções.* (E15)

### Dimensão Espiritual do Cuidado

Essa categoria teórica se revelou quando as enfermeiras obstétricas compreenderam a influência da religiosidade sobre a experiência do parto, respeitaram a manifestação das crenças das parturientes e se posicionaram em defesa da assistência livre de julgamentos.

*Cada um tem sua religião e acaba em alguns momentos transbordando* […]. *Eu procuro respeitar sempre o espaço da mulher, não me expondo como religião, porque o Estado é laico e a gente tem que respeitar o diferencial do outro, a cultura do outro… já peguei pacientes com fitinha na barriga.* […] *a gente respeita!* […] *cada profissional precisa saber lidar com essa situação, com as diferenças, com a religião e a cultura de cada um.* (E5)

*A gente tem as questões religiosas presentes na hora do parto. É de extrema importância para algumas mulheres!* […] *temos que respeitar qualquer manifestação da parte delas!* […] *percebo alguns julgamentos que não me agradam e sinalizo que a assistência precisa ser livre de julgamentos. Precisa ser igual para todos!* (E32)

## DISCUSSÃO

As adaptações implementadas pelas enfermeiras obstétricas no cuidar das parturientes, diante das medidas de controle da COVID-19, revelam a dimensão criativa do cuidado, a qual perpassou pelos seguintes elementos do processo *Clinical Caritas*: resolução criativa de problemas (sexto elemento); criação de um ambiente de cura em todos os níveis (oitavo elemento); e promoção da dignidade humana no atendimento das necessidades das mulheres no momento do parto (nono elemento).

A criatividade, compreendida como a capacidade de pensar diferente, ser inovador e criar coisas novas, associada à proatividade, que se refere à iniciativa para agir extrapolando as atividades prescritas, configuraram competências fundamentais para o trabalho em saúde durante a pandemia, as quais favoreceram a identificação de oportunidades, o enfrentamento do desconhecido e os ajustamentos ao ritmo acelerado das mudanças^(19)^.

Assim, a crise sanitária despertou a capacidade da enfermagem de se reinventar, conforme evidenciado entre as enfermeiras obstétricas deste estudo, que desenvolveram ações de cuidado em consonância com os protocolos de reorganização dos serviços de saúde no curso pandêmico, os quais sinalizavam a necessidade de evitar o compartilhamento de objetos, como os adotados para o alívio da dor, reduzir a circulação de pessoas nos serviços de saúde e manter o distanciamento físico na assistência^(2,3,4,5)^.

Assim como identificado em outras pesquisas, as enfermeiras obstétricas se mobilizaram para manter a ambiência adequada, promover conforto e bem-estar, incentivar a fisiologia da parturição e assegurar os direitos das mulheres no parto e nascimento, sem perder de vista os aspectos da segurança do processo assistencial diante das limitações impostas pelas inadequações na estrutura física dos serviços e pela escassez de recursos humanos e materiais^(1,6,9,16,20)^.

Por outro lado, essas adaptações também estão alinhadas com o sexto elemento do processo *Clinical Caritas* de Jean Watson, pois as enfermeiras aplicaram todos os conhecimentos disponíveis e utilizaram a imaginação criativa associada à lógica científica para formular propostas de solução dos problemas e, assim, fazer adaptações no processo de cuidar, recorrendo a diferentes modos de conhecer/ser/fazer/tornar-se e considerando as especificidades da pessoa em sua totalidade^(10,12,13,14)^.

Já o oitavo elemento se manifestou nas ações intencionais das enfermeiras para proporcionar um ambiente de cuidados favoráveis à confortabilidade, ao relaxamento, à privacidade e à segurança, com respeito às preferências das parturientes relativas à temperatura, iluminação e som. Tal constatação revela a preocupação das participantes com a harmonia da mente, do corpo e da alma da pessoa com o ambiente externo, compreendendo que esse alinhamento afeta positivamente a saúde física e emocional, facilitando as relações interpessoais e possibilitando uma sensação de satisfação com a vida^(10,21)^.

Ainda, no atendimento das necessidades básicas apresentadas pelas mulheres no processo de parturição, as enfermeiras demonstraram uma consciência intencionalmente cuidadosa para compreender a dinâmica das subjetividades no contexto pandêmico e resguardar a dignidade humana^(12,22)^, desvelando, assim, o nono elemento do processo *Clinical Caritas*.

Além desses aspectos, o acolhimento, o estabelecimento do vínculo e a construção de uma relação de respeito, afeto e confiança foram valorizados pelas participantes no processo de cuidar das parturientes, revelando as dimensões humanística e cultural do cuidado das enfermeiras obstétricas, as quais se mostraram ancoradas nos seguintes elementos do processo *Clinical Caritas*: manifestar valores humanístico, altruísta e ético sobre si mesma e aos outros (primeiro elemento); ter uma presença autêntica que respeita a fé, a esperança, a crença e as subjetividades (segundo elemento); empenhar-se na construção de relações de cuidado baseadas na amorosidade e confiança (quarto elemento); e permitir a expressão de sentimentos por meio da escuta autêntica do outro (quinto elemento).

O acolhimento é um processo que envolve ações para favorecer o estabelecimento do vínculo, da confiança e do compromisso entre o profissional de saúde e o usuário, propiciando a identificação das suas necessidades e o oferecimento de uma assistência humanizada e resolutiva^(12,21,22)^. No entanto, a pandemia de COVID-19 provocou mudanças significativas nas instalações físicas e nas rotinas institucionais, o que afetou diretamente os protocolos de acolhimento e humanização em prol da segurança coletiva^(23,24)^.

Nesse contexto, as enfermeiras obstétricas compreenderam que as questões sociais e emocionais inerentes à gravidez no curso pandêmico associadas à hospitalização para o parto são fatores geradores de angústia, medo e desconforto que podem influenciar significativamente a vivência do parto. Portanto, elas se mostraram empáticas e desenvolveram cuidados sensíveis e respeitosos para assegurar o vínculo, demonstrar apoio, transmitir segurança, promover bem-estar e criar as condições necessárias para o exercício da autonomia, mesmo diante das adversidades enfrentadas nos serviços obstétricos^(16,24)^.

Com base nessas concepções, as enfermeiras obstétricas flexibilizaram algumas medidas estabelecidas nos protocolos de controle da COVID-19, na tentativa de alinhar as rotinas institucionais com as necessidades e subjetividades da parturiente, assim como identificado em outros estudos^(5,7)^. Nesse sentido, por meio da observação e da sensibilidade, ofereceram cuidados para a promoção do conforto e da evolução fisiológica da parturição, tais como possibilitar a retirada da máscara pelas mulheres no período expulsivo do parto e propiciar a livre movimentação durante o trabalho de parto.

O estímulo à respiração profunda e consciente na parturição reduz a circulação de hormônios relacionados ao estresse e aumenta a liberação de endorfinas e os níveis de oxigênio, diminuindo a pressão sanguínea e tornando as sensações do trabalho de parto mais agradáveis. Ademais, a liberdade de movimentos na parturição melhora a dinâmica uterina, aumenta a tolerância à dor, otimiza a dilatação cervical e a descida da apresentação fetal, favorece a mobilidade do sacro, amplia os diâmetros ântero posterior e transverso da pelve e contribui para os puxos maternos, a oxigenação fetal e integridade perineal^(25)^.

Cabe ressaltar que, durante a pandemia, ambas práticas foram prejudicadas diante das recomendações quanto ao uso de máscara pelas parturientes nos ambientes assistenciais, pois a respiração profunda e os esforços expulsivos aumentavam a exposição às secreções respiratórias e diante da necessidade de evitar a circulação de pessoas e manter o distanciamento físico para mitigar a transmissão da COVID-19. Porém, pondera-se que a utilização da máscara de proteção pode interferir nas trocas gasosas e metabólicas inerente à fisiologia do trabalho de parto, exacerbar quadros respiratórios, comprometer a comunicação e causar sensação de desconforto e superaquecimento, ao mesmo tempo em que a proibição da livre movimentação pode figurar uma violação dos direitos das mulheres no parto^(9,16)^.

Logo, as características do processo de cuidar das enfermeiras obstétricas junto às parturientes durante a pandemia revelam convergência com o primeiro e quarto elementos do processo *Clinical Caritas*, pois o diálogo, a escuta e as demonstrações de empatia, respeito, disponibilidade, proximidade, sensibilidade e envolvimento da enfermeira oportunizam o compartilhamento de histórias de vida, desejos e angústias, colaborando para as relações de bondade-ajuda-confiança e conformando cuidados transpessoais voltados para a promoção do conforto, do bem-estar e da recuperação da pessoa cuidada. Sob essa ótica, as enfermeiras entram na experiência do cuidado e vice-versa, conectando-se e despertando-se para os problemas do outro de forma que transcende o físico^(10,12,13,14,26)^.

A partir dessa perspectiva, foram evidenciados o segundo e terceiro elementos do processo, visto que as participantes deste estudo: manifestaram a consciência intencional e a presença autêntica; mantiveram-se atentas às subjetividades; reconheceram as mulheres em sua totalidade; perceberam seus movimentos e significados; possibilitaram a livre expressão de pensamentos e sentimentos; foram respeitosas e compreensivas; e incentivaram a confiança e a esperança em um cenário de medo e incerteza generalizada^(10,26,27)^.

Permeando o cuidar das parturientes no curso pandêmico, salienta-se a dimensão educativa do cuidado das participantes, as quais se mostraram genuinamente engajadas com ações de ensino e aprendizagem. Assim, o cuidado agregou características transpessoais, pois, a partir do encontro, a enfermeira estabelece uma relação intersubjetiva que exige autenticidade, estar reflexivamente presente para si e para o outro e agir com uma consciência intencional para promover troca de saberes, vivências e significados, além de capturar a realidade da pessoa, perceber como ela compreende e responde às circunstâncias de um determinado momento^(10,12,13,14)^.

Nesse percurso concernente ao décimo elemento do processo *Clinical Caritas*, a enfermeira estabelece um relacionamento empático e reconhece o direito de escolha da pessoa; por isso, considera seus saberes, suas experiências e necessidades, fornece informações apropriadas, esclarece dúvidas e incentiva sua participação nos processos decisórios, demonstrando respeito aos seus referenciais de crenças e valores^(11,14)^. Sob essa ótica, as enfermeiras obstétricas oferecem orientações e opções de cuidado, pois compreendem que o diálogo oportuniza adaptações do processo de cuidar à realidade cultural da parturiente, proporcionando uma assistência respeitosa que gera a confiança e favorece a adesão aos cuidados propostos^(25)^, conforme identificado entre as participantes desta pesquisa.

Sabe-se que, durante a pandemia de COVID-19, a proposta dialógica das atividades de educação em saúde desenvolvidas pelas enfermeiras foi fundamental para disseminar informações baseadas na ciência, incentivar comportamentos seguros, promover o autocuidado e minimizar o sofrimento relacionado aos altos números de contaminação e morte pela doença^(28)^. Nesse contexto, a religiosidade e a espiritualidade também foram recursos de enfrentamento utilizados por muitas pessoas, uma vez que estimulam o cuidado, a generosidade, a empatia, a solidariedade e a resiliência como expressões da vida baseadas nos princípios humanísticos^(29)^.

Cabe esclarecer que a religiosidade trata de rituais e cerimônias relacionadas ao transcendente, praticadas em ambiente privado ou público, contemplando crenças específicas sobre a vida, a morte, os papéis e as condutas no contexto de determinados grupos sociais. Por outro lado, a espiritualidade se refere à busca pessoal pelo sentido da vida e da sua relação com o sagrado e o transcendente, que possibilita a transformação da realidade da pessoa, independentemente da associação com uma religião ou comunidade^(30)^. Nesse sentido, ambas têm relação com o aumento da qualidade de vida, a melhoria da função imunológica e do vigor em pessoas com doenças crônicas, a redução do estresse relacionado às patologias degenerativas e neurológicas, bem como do tempo de hospitalização^(29,30)^.

A partir desse reconhecimento, nota-se que o processo de cuidar das enfermeiras obstétricas contemplou uma dimensão espiritual, que auxilia na autodescoberta, na manutenção da saúde e no enfrentamento de situações consideradas difíceis^(10,14)^. Sob essa ótica, identifica-se o terceiro elemento do processo *Clinical Caritas*, que se traduz na sensibilidade e no comprometimento da enfermeira com o sistema de fé-esperança da pessoa e no oferecimento de cuidados que visam ao restabelecimento do equilíbrio vital, ao resgate dos sentidos da vida e à autorrealização. Concomitantemente, apresentou-se o décimo elemento, relativo à ampliação da visão da enfermeira para os fenômenos espirituais e desconhecidos, possibilitando a preservação dos significados, das crenças, dos mitos, das metáforas e do mundo da vida interior e subjetiva, por meio de cuidados que abarcam a totalidade do ser e transcendem a linearidade do fazer^(10,12,13,14,27)^.

Diante do exposto, cabe destacar que os aspectos abstratos que compõem a proposta de cuidado transpessoal de Jean Watson e o fato de se tratar de uma teoria de grande alcance podem dificultar sua aplicação na prática da enfermagem^(14,21)^. No entanto, ainda que as enfermeiras obstétricas deste estudo não tenham utilizado a referida teoria para sistematizar o seu cuidar em meio às adversidades da pandemia de COVID-19, foi possível perceber que as dimensões do cuidado dessas especialistas apresentam aspectos que remetem aos dez elementos do processo *Clinical Caritas*.

Como limitações do estudo, aponta-se a técnica adotada para a captação das enfermeiras obstétricas, o que pode ter resultado em uma certa homogeneidade das participantes, as quais atuavam em serviços públicos de atenção ao parto e nascimento do Rio de Janeiro. Por isso, os achados desta pesquisa não são representativos da realidade vivida por enfermeiras de outras localidades brasileiras durante o contexto pandêmico.

## CONCLUSÃO

Evidencia-se que, durante a pandemia de COVID-19, o cuidado das enfermeiras obstétricas às parturientes apresentou dimensões que perpassaram os dez elementos do processo *Clinical Caritas* de Jean Watson. Portanto, elas conseguiram acessar aspectos subjetivos das parturientes para efetivar a transpessoalidade no cuidado, auxiliando-as no alcance de um grau mais elevado de harmonia, em um ambiente onde as medidas de controle da COVID-19 implementadas nos serviços de saúde conduziam a uma assistência procedimental, prescritiva, curativa e impessoal. Esses achados apontam para a relevância da atuação dessas especialistas na promoção da humanização e das boas práticas mesmo em cenários de crise sanitária, sendo necessários estudos que explorem a perspectiva das mulheres acerca da atenção ao parto e nascimento nesse contexto.
